# Analysis of Social Policy and the Effect of Career Advancement Support Programs for Female Doctors

**DOI:** 10.1089/whr.2021.0038

**Published:** 2021-08-19

**Authors:** Kae Okoshi, Kayo Fukami, Yasuko Tomizawa

**Affiliations:** ^1^ Department of Surgery, The Japan Baptist Hospital, Kyoto, Japan.; ^2^ Research Center for Science, Technology and Social Communication in Next Generation, Doshisha University, Kyoto, Japan.; ^3^ General Education, National Institute of Technology, Toba College, Mie, Japan.; ^4^ Department of Cardiovascular Surgery, Tokyo Women's Medical University, Tokyo, Japan.; ^5^ Kyoritsu Christian Institute, Tokyo Christian University, Chiba, Japan.

**Keywords:** career advancement, female doctors, female workforce participation, gender equality support program, social policy

## Abstract

***Background:*** In Japan, the number of female doctors has gradually increased; however, they form less than half of the average percentage (46.3% in 2016) among the Organisation for Economic Cooperation and Development member countries. In addition, some female doctors reduce their working hours for childbirth, housework, and childcare. Thus, women find it challenging to continue medical practice in Japan. The Ministry of Education, Culture, Sports, Science, and Technology (MEXT) established a time-bound grants program from 2007 to 2009 to support female doctors and improve their working environment. This study examines the program contents and the increase in female doctors in university hospitals.

***Materials and Methods:*** Using individual data from the *Survey of Physicians*, *Dentists*, *and Pharmacists* from 1996 to 2016, we compared two categories of female doctors, faculty and nonfaculty members, at university hospitals that received grants compared to those that did not. In addition, we reviewed the support program for female doctors and nurses developed by nine university hospitals using content from the MEXT and information from previous studies.

***Results:*** Most programs included in-hospital childcare and shorter working hours. There were fewer women in the nine hospitals receiving grants compared to other university hospitals. There were significant differences in the percentages of male and female nonfaculty members in 2000, 2004, and 2008.

***Conclusions:*** While we could not find any evidence that programs supported by the grants could increase female doctor numbers, these programs may have improved the status of female doctors with children. More intensive measures are needed to increase the number of women doctors in leadership positions.

## Introduction

As of 2018, there were 71,758 female doctors in Japan, representing only 21.9% of all doctors in Japan.^1^ Although the ratio of female doctors has gradually improved since the 1970s (when they accounted for less than 10% of all the medical doctors nationwide^2^), the percentage of female physicians remains less than half of the average percentage (46.3% in 2016) among Organisation for Economic Cooperation and Development member countries.^3^ The Japanese government has recently promoted women's participation in paid work by enacting the Act on Promotion of Women's Participation and Advancement in the Workplace.^4^ The government hopes to raise the ratio of women at work in Japan to match other developed countries. Despite these promotions, the rate of increase in the percentage of female doctors has remained slow in recent years, reflecting the challenges of women's participation in medical practice in Japan.

One of the key factors lowering the percentage of female doctors in Japan is that they often leave the profession around 11 years after graduation due to life events, such as pregnancy and childbirth.^1^ Those female doctors who quit their jobs gradually return after a few years. However, many work fewer hours on returning due to housework and parental duties.^1^ They work part-time and are exempted from night shifts and on-call duties. The Ministry of Health, Labour and Welfare (MHLW) estimated that a female doctor's working hours are 80% of that of a male doctor.^5^

Thus, a need has arisen to support female doctors in continuing to work after pregnancy and childbirth. Since 2005, the Ministry of Education, Culture, Sports, Science, and Technology (MEXT) has publicly implemented a Grant for Social Needs, including Regional Medicine, and each year, suggests and selects various themes. The MEXT decided to provide grants to nine university hospitals to develop a Support Program for Promoting the Stability and Return of Female Doctors and Nurses to Clinical Sites (hereinafter referred to as “the support program for female doctors and nurses”^6^). In 2007, out of 42 applications, 9 university hospitals were selected for the grant.^6^ These programs have since been implemented for 3 years (2007–2009) to support female doctors and nurses in settling in and returning to clinical practice from maternity or parental leave. The applicants selected for “the support program for female doctors and nurses” ran their educational programs from 2007 to 2009 using the grant fund.

“The support program for female doctors and nurses” was one of the government's first grants to support female doctors and is regarded as the forerunner of subsequent support for female doctors. These programs included establishing and expanding in-hospital preschools and childcare facilities for sick children of staff and creating new full-time positions with shorter work timings. Furthermore, it included supporting their return to work, holding workshops and symposiums on career education for female doctors, and establishing a remote diagnosis system, which allowed female doctors to work from home.^7^ Following “the support program for female doctors and nurses,” similar supportive programs for female doctors have been implemented in some other hospitals in Japan.

It is important to note that support for female doctors in Japan has often focused on supporting childcare and reducing working hours.^8,9^ However, in other countries, support for female doctors consists of family-friendly workplaces and a wider range of career support, such as mentorship programs, professional networking, and programs aimed at leadership development.^10^ Of these, the effectiveness of mentorship programs has been particularly well reviewed,^11^ leading to an increase in the number of female faculties,^12^ a high level of satisfaction,^13^ and no difference in academic activity between genders.^14^ This indicates that it is important to evaluate the effectiveness of support programs for female doctors in Japan.

To design evidence-based career development programs for female doctors in the years ahead, it is crucial to know which career support programs are effective. However, to the best of our knowledge, very limited previous research has quantitatively assessed this topic.

In this study, we review the grant amounts provided to nine university hospitals in Japan and their program details to support female doctors. In addition, based on individual data from the *Survey of Physicians*, *Dentists*, *and Pharmacists* (SPDP) from 1996 to 2016, we compare the percentages of two categories of female doctors, faculty and nonfaculty members, at university hospitals that received the grants with those that did not. Finally, we discuss the effectiveness of the programs based on these analyses.

## Materials and Methods

### Sample and data collection methods

Out of 42 universities that applied, 9 were accepted for “the support program for female doctors and nurses” by MEXT, and we reviewed those 9 programs. The authors obtained each program's title and contents from the MEXT website^6^ and previous studies reporting on each program. The authors received specific grant amount details for each university through email communication with MEXT.

The study also included anonymized individual data from the SPDP from 1996 to 2016. This survey is conducted every 2 years by the government. The authors received permission from the MHLW to use the database (Statistics Law Article 33). While all licensed physicians, dentists, and pharmacists in Japan are obligated to register for the survey, the authors only obtained data related to physicians. The Ethics Committee of National Institute of Technology, Toba College stated that this study did not need to be reviewed ethically. The individual data included gender, prefecture of work, and type of work (*e.g.*, university, public or private hospital, clinic, or research institute). Although the names of the institutions were anonymized in the SPDP, only one of the nine university hospitals was located in each prefecture; thus, the authors could select those who worked at university hospitals according to the prefecture and type of work. The MHLW provided permission to release data from the SPDP on the condition that the authors do not disclose the names of individual universities.

### Statistical analyses

Pearson's chi-square test was performed to compare the proportion of male and female doctors (nonfaculty and faculty) in nine university hospitals and the other university hospitals (71 from 1996 to 2014 and 72 in 2016, as Tohoku Medical and Pharmaceutical University was newly established) using JMP version 5 (SAS Institute, Inc., Cary, USA).

## Results

### Program description

The names of nine universities selected for “the support program for female doctors and nurses,” program titles, contents with references, and the total grant amounts for the 3 years from 2007 to 2009 are presented in Table 1. The grant amounts were converted from Japanese yen to US dollars based on purchasing power parity from 2007 to 2009.^15^ The nine universities selected for the program were Asahikawa Medical University, Tsukuba University, Kobe University, Shimane University, Okayama University, Kyushu University, Osaka City University, Wakayama Medical University, and Jichi Medical University. The individual programs of each university are discussed below.

**Table 1.** Details of the Nine University Hospitals Selected for “*the Support Program for Female Doctors and Nurses*,” Their Program Details, and Grant Amounts

University	Title	Contents	Grant for 3 years (USD 1000)	References
Asahikawa Medical University	Plan to support child and elderly care throughout the hospital	Matching doctors on leave to return to work, providing home study systems, establishing short-time work systems, providing childcare for sick children, holding seminars and lectures	538	^16,17^
Tsukuba University	Career support system for women doctors and nurses	Creating training programs, career counseling, e-learning systems, shortened working hours for full-time employees, organizing seminars and symposiums	536	^18,19^
Kobe University	Systematic development brush-up education for doctors and nurses: Development of Internet programs and catch-up programs for female doctors and nurses to return to work	Holding online courses, e-learning, video conference maintenance	430	^20–22^
Shimane University	New model project for career continuation—Aiming for a flexible career of female medical professionals	Providing career education and counseling services, childcare support services in conjunction with the hospital's nursery, and developing web-based home learning and remote diagnosis systems	535	^23^
Okayama University	Program for Supporting Female Physicians for their Continuing Career Development	Introduction of advisors, education by simulation, shortened workday, sick childcare, formation of male supporters' club	492	^24–26^
Kyushu University	Women Medical Professional Support Program “KIRAMEKI Project”	Shortened working hours for female doctors with young children, establishment of outpatient clinic for female patients by female doctors, survey on female doctors, setting up a website, making e-learning contents, holding gender education and lectures	534	^27,28^
Osaka City University	Aiming for both parental and career advancement	Organizing symposiums, shortened working hours for female doctors with young children, establishing a sick childcare center, and providing e-learning materials	519	^29^
Wakayama Medical University	Support for female doctors to return to work after childbirth and parental leave	Development of on-campus nursery, sick children's care, and evening care, holding forums and seminars	536	^30–32^
Jichi Medical University	Female Doctor Support Program of Jichi Medical University	Holding lectures, setting up a website, conducting seminars and workshops, shortened working hours for female doctors with young children, providing individual consultations	517	^33,34^

Asahikawa Medical University (program name: Plan to support child and elderly care throughout the hospital^16^) established the “Nirinso Center” as an operational department to provide childcare support and nursing care support for older adults. During the program, the center provided doctors and nurses looking for work after taking leave for childbirth, childcare, or nursing care with the information they needed to return to work, as well as support for self-education. Furthermore, although the short-time working benefit is usually not offered to workers who have worked for less than 1 year, this program allowed it. In addition, the center provided a childcare facility for sick children, collaborated with the 24-hour preschool, and established a liaison with the public childcare support services for Asahikawa citizens. Various projects, such as seminars, lectures, workshops, surveys, meetings for exchanging opinions, and children's day camps, were carried out.^16,17^

The University of Tsukuba (program name: Career support system for women doctors and nurses^18^) implemented a program that focused on counseling and career advancement to support return to work. Fourteen female doctors and eight nurses received support over 3 years. The support was divided into three categories: consultation and career counseling, training coordination, and environmental improvement. Regular interviews were conducted to provide consultation on career planning and mental health care and support for reemployment. As training coordination, training programs were created, applicants were matched with medical institutions, and educational materials and programs were developed. For environmental improvement, the program included introducing a full-time/part-time system, a training dispatch system, and information on using the preschool in the university. Seminars, get-togethers, and opinion-exchange meetings were also held.^18,19^

Kobe University (program name: Systematic development brush-up education for doctors and nurses: Development of Internet programs and catch-up programs for female doctors and nurses to return to work^20^) established the “D&N (doctors and nurses) Brush-Up Center” in its hospital. This aimed to support female doctors and nurses on pregnancy and maternity leave in improving their skills and attempted to invigorate their return to clinical practice by providing online courses and a conference participation system for each department.^20,21^ At the same time, Kobe University was selected as a recipient of the Special Coordination Funds for Promoting Science and Technology from the Japan Science and Technology Agency for the “Development of a Model Female Researcher Support Program.”^22^ Running parallel to the “Systematic Development of D&N Brush-up Education,” the “Development of a Model Female Researcher Support Program” comprehensively supported female physicians and researchers; female doctors and researchers may have benefited from both.

Shimane University (program name: New model project for career continuation—Aiming for a flexible career of female medical professionals^23^) launched the Office of Female Staff Support to provide a comprehensive environment for female doctors. This included career education and consultation service, a childcare support program in conjunction with the hospital's preschool, and a skill enhancement support program through the development and operation of a web-based home-based learning and remote diagnosis system. After the completion of “the support program for female doctors and nurses,” the office was renamed the “Work–Life Balance Support Office.”

The program at Okayama University (program name: Program for Supporting Female Physicians for their Continuing Career Development^24^) was based on a three-pronged approach. The “Optimal Advisor Referral System (commonly called Okayama MUSCAT)” aimed to establish female doctors in the clinical field, and the “Family-Friendly Return to Work Course (commonly called MUSCAT WILL)” supported the return of female doctors to work. In addition, increasing their families' comprehension and cooperation, as well as that of men, in the workplace helped to facilitate the abovementioned main approaches.^25^ Due to these efforts, a childcare facility was established for hospital staff members' sick children, and 37 doctors returned to work through “MUSCAT WILL.”^26^ After the completion of “the support program for female doctors and nurses,” the program has been continued as the “MUSCAT Project” operated by the Okayama Prefecture Women's Doctor Career Center.^24^

Kyushu University's program (program name: Kirameki Project for medical professionals^27^) established the “Kyushu University Center for Education and Research of Women Medical Personnel.” This program helped build an e-learning system, held a seminar on gender issues for medical students, as well as a course on gender-based medicine, and conducted various lectures and seminars.^28^ After completing the program, it continued as the “Kyushu University Hospital Kirameki Project” with its budget until March 2019. From April 2019, it continued as the “Kirameki Project” with the inclusion of clinical education and training projects and initial and late training for doctors and dentists as part of the regular training.^27^

In 2007, Osaka City University (program name: Aiming for both parental responsibilities and career advancement^29^) established the “Support Center for Female Doctors and Nurses.” The center conducted publicity and awareness activities through symposiums and other events. It implemented short working hours and established and operated a child-care facility for sick children, held symposiums on regional cooperation systems, and provided outplacement support through the development and provision of e-learning materials. The program has continued to be implemented under the same name by the university.

Wakayama Medical University (program name: Support for female doctors to return to work after childbirth and parental leave^30^) established the Women's Medical Personnel Support Center and later expanded it to the Work–Life Balance Support Center in 2017.^31^ The center provided information on systems to support childbirth and childcare, operated and managed a preschool, and supported career continuity,^32^ but it was unclear whether these activities and services were conducted and provided during or after the program.

Jichi Medical College (program name: Female Doctor Support Program of Jichi Medical University^33^) established the Center for Female Medical Doctors to support the development of the next generation of doctors, continued employment, return to work, and childcare. Furthermore, the program supported the development of community health care workers. To support continued employment, seminars and workshops were held, a shorter working hours system was established, and individual consultations were provided.^34^ After the completion of the program, it was reorganized as an independent project of the affiliated hospital as the “Jichi Medical College Physician and Researcher Career Support Center” in 2010 and started sick childcare and nighttime care services for a wider range of hospital staff.^33^

### Gender distribution in employment

Table 2 presents the gender distribution of respondents of the SPDP from 1996 to 2018. It further includes the percentage of women respondents. In the 22 years between 1996 and 2018, the percentage of female doctors increased from 13.4% to 21.9%.

**Table 2.** The Number of Respondents and Their Percentages as Presented in the *Survey of Physicians*, *Dentists*, *and Pharmacists from 1996 to 2018*

Year of the survey	No. of respondents	Men	Women	Percentage of women
1996	240,908	208,649	32,259	13.4
1998	248,611	213,603	35,008	14.1
2000	255,792	218,940	36,852	14.4
2002	262,687	221,548	41,139	15.7
2004	270,371	225,743	44,628	16.5
2006	277,927	229,998	47,929	17.2
2008	286,699	234,702	51,997	18.1
2010	295,049	239,152	55,897	18.9
2012	303,268	243,627	59,641	19.7
2014	311,205	247,701	63,504	20.4
2016	319,480	251,987	67,493	21.1
2018	327,210	255,452	71,758	21.9

### Comparative employment status

**FIG. 1.**
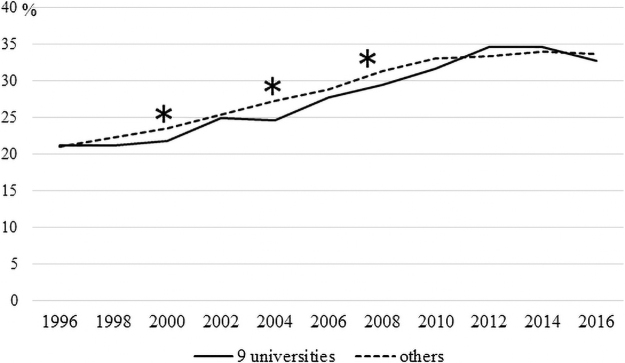
The percentage of female doctors (nonfaculty members) working at the nine university hospitals selected for “the support program for female doctors and nurses” compared to those in other university hospitals. *p-value <0.05.

**FIG. 2.**
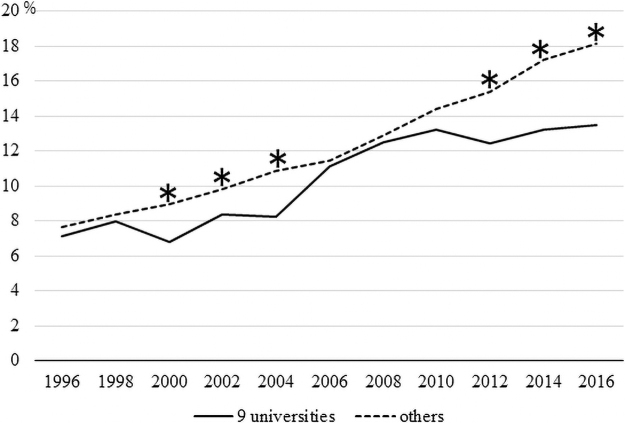
The percentage of female doctors (faculty members) working at the nine university hospitals selected for “the support program for female doctors and nurses” compared to those in other university hospitals. *p-value <0.05.

Figures 1 and 2 illustrate the percentage of female doctors (divided into nonfaculty and faculty members) in the nine university hospitals selected for “the support program for female doctors and nurses” compared to those in other university hospitals.

There were significant differences in the percentages of male and female nonfaculty members in 2000, 2004, and 2008 (*p* = 0.0031, *p* = 0.0019, *p* = 0.0437, respectively; Fig. 1). There were fewer women in the nine university hospitals than those in the other university hospitals. There were statistically significant differences in the percentages of male and female faculty members in 2000, 2002, 2004, 2012, 2014, and 2016 (*p* = 0.0017, *p* = 0.0379, *p* = 0.0004, *p* < 0.0001, *p* < 0.0001, *p* < 0.0001, respectively; Fig. 2). There were fewer women in the nine university hospitals compared to those in other university hospitals.

## Discussion

The number of female faculty members in medical schools is especially low.^35^ The lack of role models seems to hinder women further; increasing the number of female faculty members is key in encouraging the next generation of female doctors.^36^ Figures 1 and 2 illustrate that the percentage of female doctors in both the nonfaculty and faculty roles in universities where supportive programs were not running tended to be higher. Figure 2 in particular shows that the percentage of female doctors in universities other than the nine universities examined in this study has been higher since 2012. Therefore, it is difficult to say whether “the support program for female doctors and nurses” has contributed to an increase in the number of practicing female doctors. However, in Figure 2, the percentage of female faculty members in the nine universities is quite close to the percentage in other universities from around 2006 to 2010. This result could indicate growing momentum within universities that implemented the program to support female doctors, although the effect of the program on the increasing number of increasing female doctors would not have been sustained.

Figure 1 illustrates that the percentage of female nonfaculty members in the nine grant-funded universities examined in this study temporarily exceeded that of the other universities in 2012 and 2014. Most of “the support program for female doctors and nurses” were designed to help female doctors balance work and childcare, return to work, and stay connected to clinical practice, even if only for a short time. While these programs might not have had an immediate effect on helping female senior doctors in leadership positions, such as faculty members, they might have made it easier for junior female doctors (especially those with small children, *e.g.*, those targeted by the program) to remain in or return to the workplace.

A common feature of all the programs was the establishment of a department in charge of the program. This department conducts educational activities both inside and outside the university, such as seminars and lectures. It continues to provide and develop support systems in some form, even after the end of the grant period. In particular, face-to-face events are held very frequently at all the universities. The scale of these events ranges from on-campus gatherings to large-scale symposiums and exchange of opinions with other universities. The program coordinators and staff worked actively on these events. Publicity posters on campus and websites were used to promote the program, which might have had an educational effect.

Many of these universities had experience supporting the careers of female doctors, researchers, and nurses in some way before the implementation of “the support program for female doctors and nurses.” In some cases, they had received grants from other sources concurrently with this program. For example, most university hospitals have in-hospital childcare and shorter working hours, but it is often difficult to demonstrate whether this was implemented because of the current program or the completion of a previous program. This feature made it difficult to analyze the program's effect. However, this difficulty in determining the impact of the current program is evidenced by the fact that the nine universities selected for the program have traditionally taken an interest in supporting the careers of female physicians and have invested personnel in multiple support projects. Recipients of resources implemented during this program (*e.g.*, e-learning, lectures, seminars, and symposiums) were assembled in a short period. The program contributed to this outcome and played a role in the further development and expansion of the environment after the grant-supported program.

The program focused on both female doctors and nurses' careers; however, their lifestyles are vastly different. We would like to point out that even though childcare and elderly care are the responsibility of both genders, both “the support program for female doctors and nurses” and universities' programs have focused only on women. This reveals the assumption that men do not take care of their children and could perpetuate the patriarchal mindset that only women are responsible for childcare. This is contrary to the current values of gender equality. In addition, shortened hours and exemption from night duty might be unavoidable when the child is an infant, but they increase the workload and income gap between men and women in the long term. Japanese women are generally expected to assume most domestic responsibilities, while the work environment remains male dominated.^37^ This scenario holds for doctors as well. In 2018, it was revealed that female applicants to medical schools in Japan had been unfairly discriminated against by manipulation of their examination scores.^38^ A MEXT investigation found that women might have been discriminated against at four medical schools. The head of one of the medical schools attributed this discriminatory measure to the tendency of female doctors to work fewer hours than male doctors. Such discriminatory practices appear to be influenced by the outdated and prevalent value system in Japanese society that doctors must be male. In fact, a popular Japanese magazine claimed that female doctors were unsuitable as surgeons based on the claim that they are physically and mentally weak and that gender control is necessary.^39^ Thus, while overcoming these archaic concepts may seem futile, it is necessary to change them through the implementation of such programs.

The MHLW estimated that a female doctor's working hours are 80% of those of a male doctor.^5^ A previous study reported that in the surgical field, men and women worked for 91.5 and 76.1 hours/week, respectively.^40^ This disparity might be due to fixed gender roles, with men working excessive hours and having little involvement in their families.^41^ For example, Japanese female surgeons with children spend approximately three extra hours a day on household chores and parental duties than their male counterparts.^42^ Widening the gap between the working hours of women with children and the rest of the workforce could increase the burden on the latter, who might find it frustrating to have to work longer hours or more night shifts. While it may be inevitable for mothers to temporarily choose to work shorter hours when their children are infants, the system should also make it easier for fathers to choose reduced working hours for childcare.^43^ Other issues that still need to be addressed are related to female doctors' gradual return from shorter to longer working hours and from night-duty exemptions to full-time duty. It is assumed that systems and expertise for childcare support have been established through these support programs. In the future, specific numerical goals and measures are needed to determine how female doctors, who can now work in university hospitals as a result of these support programs, can achieve success in their respective fields of clinical practice, research, and education.

In this study, the authors were unable to prove that “the support program for female doctors and nurses” increased the number of female doctors in faculty or nonfaculty positions.

Future studies focused on increasing the number of female doctors, especially in leadership positions, are required. It may be difficult to increase the percentage of female doctors over several years of the programs without numerical goals. However, implementing these programs over several years may have helped raise the status of female doctors who are pregnant or raising young children and played a role in reducing inequality after the programs. Although these programs did not result in an increase in the percentage of female doctors within the period of the study, the authors expect that it will become a matter of course for female doctors to continue working as doctors while using in-hospital childcare support.^8^

Previously, barriers to career development for female surgeons were shown to be related to the absence of residency/fellowship support, mentorship/sponsorship, leadership, work–life balance, and pay equity.^44^ Therefore, for female doctors to aim for leadership positions and boost their clinical and academic achievements, active measures may be required, such as mentoring and networking by experienced female leaders, encouraging the acquisition of competitive research grants, and setting qualitative criteria for promotion.^10^

A recent systematic review of women's leadership in academic medicine found that leadership development programs had a positive impact on women's leadership development and medical school culture.^45^ There are few female board members and councilors in medical societies in Japan.^46^ While some medical societies are making efforts to increase their numbers of female members, the proportion remains small.^47^ Moreover, it is difficult to increase the number of female board members and councilors if, as in the case of the traditional selection process, they are to be selected from among the representatives of institutions. There may be different opinions regarding the increase in the quota for women, but we consider it appropriate that this quota at least matches the ratio of women who hold academic society membership. In addition, as for female faculty members at universities, female board members and councilors in each society can serve as important role models for young female doctors, and they are more likely to make decisions that do not disadvantage women when implementing the society's policies, programs for society meetings, and programs for medical specialists. In addition, it is essential to monitor the inclusion of women in professional societies, because leaders of professional societies tend to have a significant influence on scholarly inquiry and research in their respective fields.^48^ However, alongside the implementation of these measures and programs that have proven to be effective in studies conducted outside Japan, it is necessary also to recognize and deliberately correct deep-rooted and continuous gender discrimination that arises from conscious or unconscious bias.^44^ Accordingly, support for building female doctors' careers should not remain limited to childcare support.

## Conclusion

In conclusion, we could not find any evidence that “the support program for female doctors and nurses” could increase the number of female doctors, whether faculty or nonfaculty members. The percentage of female faculty members and nonfaculty members was lower in university hospitals that implemented “the support program for female doctors and nurses” than in other university hospitals.

Our study has several limitations. First, although the study compared the percentage of female doctors at the nine university hospitals with other university hospitals, the authors did not examine the effects of individual programs. There may be other reasons for the increase in the female doctor employment rate at each of the nine university hospitals. Second, several universities had experience in providing some form of career support to women before the implementation of the program, and in some cases, received grants from other sources concurrently with “the support program for female doctors and nurses.” This feature made it difficult to analyze the program's effectiveness, which was the purpose of this study. For example, although most university hospitals had in-hospital childcare and shorter working hours, it was often difficult to determine whether they had been implemented because of the program or whether they were pre-existing. However, it is reasonable to assume that the program was a catalyst for accelerating support measures. Furthermore, the contents of “the support program for female doctors and nurses” are so wide ranging that it is difficult to verify their effectiveness.

Nonetheless, this is the first study to directly evaluate the effectiveness of “the support program for female doctors and nurses” in Japan. Further funding and efforts are required for supporting programs that demonstrate high effectiveness based on evidence. It is important to reflect on the evaluations of these programs. Moreover, more aggressive approaches are required, such as mentoring and networking by experienced female leaders, encouraging the acquisition of competitive research grants, and setting qualitative criteria for promotion to increase the number of female doctors, especially in leadership positions.

## Abbreviations Used

MHLW: Ministry of Health, Labour and Welfare
MEXT: Ministry of Education, Culture, Sports, Science, and Technology
SPDP: *Survey of Physicians*, *Dentists*, *and Pharmacists*
